# Construction of gene subgroups of Crohn disease based on transcriptome data

**DOI:** 10.1097/MD.0000000000034482

**Published:** 2023-08-04

**Authors:** Jianglei Ma, Huijie Zhang, Yuanyuan Zhang, Guangming Wang

**Affiliations:** a School of Clinical Medicine, Dali University, Dali, China; b Department of Obstetrics, The Affiliated Yantai Yuhuangding Hospital of Qingdao University, Yantai, China; c Genetic Testing Center, The First Affiliated Hospital of Dali University, Dali, China.

**Keywords:** bioinformatics, Crohn disease, gene identification, subpopulation

## Abstract

The global prevalence of Crohn disease (CD), a chronic inflammatory disease of the intestine, has been increasing; however, the etiology and pathogenesis of this disease have not been fully elucidated. Therefore, in the present study, we aimed to better understand the molecular mechanisms underlying CD to aid the development of novel therapeutic strategies for this condition.

**Methods:**

Based on the transcriptome data from patients with CD, this study used an unsupervised learning method to construct gene co-expression molecular subgroups and the R and SPSS software to identify the biological, clinical, and genetic characteristics and signatures of each subgroup.

**Results:**

Two subgroups were analyzed. Compared to subgroup II, subgroup I consisted of older patients with a more limited range of disease presentation and had a higher number of smokers. The specific genes associated with this subgroup, including *CDKN2B*, solute carrier family 22 member 5, and phytanoyl-CoA 2-hydroxylase, can be targeted for managing intestinal dysbacteriosis. The number of patients showing infiltrating lesions was higher, the number of smokers was lower, and CD severity was worse in patients in subgroup II than those in subgroup I. The specific genes relevant to subgroup II included cluster of differentiation 44, tryptophanyl-tRNA synthetase, and interleukin 10 receptor, alpha subunit, which may be related to viral infection.

**Conclusion:**

The present study segregated patients with CD into 2 subgroups; the findings reported herein provide a new theoretical basis for the diagnosis and treatment of CD and could aid a thorough identification of potential therapeutic targets for this disease.

## 1. Introduction

Crohn disease (CD) is a chronic intestinal inflammatory disease with typical clinical manifestations of abdominal pain, diarrhea, weight loss, and fatigue. The incidence of CD is growing in many regions worldwide and is higher in high-income countries than in low- and middle-income countries. Between 1930 and 2008, Canada and Europe reported the highest incidence rate of CD (20 per 100,000 individuals per year average and 322 per 100,000 individuals per year average, respectively).^[1]^ The age of onset of CD is mainly 20 to 40 and 50 to 60 years.^[2]^ However, the etiology of CD is unclear, and its pathogenesis has not been fully clarified. Currently, it is known that complex interactions among various factors, such as genetics, the environment, and intestinal microbiota disorders, cause CD.^[3]^ The disease can cause congenital and adaptive immune response disorders, which frequently result in defects in epithelial barrier function and T cell apoptosis.^[4,5]^ Studies show that the median time from symptom onset to diagnosis in patients with CD is 8.3 to 29.9 months.^[6]^ Late diagnosis delays disease treatment and increases the risk of complications.

Early diagnosis and treatment improve the chances of good therapeutic outcomes. The Paris expert consensus defines early CD as the stage in which there are no complications and no drugs affecting the disease course within 18 months of diagnosis.^[7]^ However, as the time between symptom onset and diagnosis differs between patients, the effect of similar treatments during the early CD stage may also vary, raising questions as to the utility of this categorization. Some researchers have used anti-*Saccharomyces cerevisiae* antibody as a potential serological marker for CD diagnosis, but anti-*Saccharomyces cerevisiae* antibody positivity accounts for only 35% to 60% of patients with CD.^[8]^

There exists no treatment for CD that results in a complete cure. Traditional drugs, such as 5-aminosalicylic acid, glucocorticoids, and immunosuppressants, can temporarily control and relieve symptoms, but adverse reactions are common, and relapse frequently occurs after drug withdrawal.^[9]^ With the development of biologically active molecules, biological agents, such as anti-tumor necrosis factor-α monoclonal antibodies, have shown promising effects in the treatment of CD.^[10]^ Owing to individual differences, the use of anti-tumor necrosis factor-α antibodies may not be effective in all patients. In Ontario, Canada, the 10-year public expenditure on monoclonal antibodies for CD patients ran 3 times over budget but has not resulted in a significant decrease in the CD-related hospitalization or bowel resection rate.^[11]^ Therefore, exploring CD pathogenesis-related genes would be helpful for guiding clinical diagnosis and treatment.

Therefore, in this study, we aimed to better understand the molecular mechanisms underlying CD and aid in the development of new therapeutic strategies. To the best of our knowledge, this study is the first to report on the construction of molecular relevant CD subgroups using bioinformatics. In this study, we grouped transcriptome data from 1156 patients with CD and 201 control patients and used bioinformatics methods to determine the characteristics and reliability of these subgroups. Notably, this study provides molecular evidence for the discovery of new therapeutic targets in CD.

## 2. Materials and methods

### 2.1. Gene Expression Omnibus (GEO) data download and processing

The GEO database (https://www.ncbi.nlm.nih.gov/geo/) provides free high-throughput functional genome datasets. “Crohn’s disease” was entered into the search interface, and datasets GSE112366, GSE186582, GSE207022, GSE20881, GSE60083, and GSE95095 and their corresponding platform files GPL13158, GPL570, GPL13158, GPL1708, GLP6884, and GPL14951 were downloaded; these sets only contained patients with CD and control group patients. The “limma” (v.3.54.2) and “sva” (v.3.46.0) packages in the R software (v4.2.2, 64 bit; https://www.r-project.org/) were used to preprocess the datasets, eliminate the batch effect, and obtain a unified dataset.^[12,13]^ As the data in the GEO database are public and do not include patients’ personal information, the patients’ ethical approval and informed consent were not required.

### 2.2. Establishment of molecular subgroups

The “ConsensusClusterPlus” (v.1.62.0) package in the R software (v4.2.2; https://www.bioconductor.org/) was used to cluster the genes of 1156 patients with CD through an unsupervised learning method.^[14]^ This method divides genes with similar genetic characteristics into 2 to 10 clusters by quantitatively and visually estimating the number of unsupervised clusters in a dataset. Based on the cluster consistency score, the cluster with the highest score for each subgroup was selected for the grouping.

### 2.3. Analysis of clinical characteristics of subgroups

The patients’ sex, age, smoking status, presence of infiltration in the lesion, and Simple Endoscopic Score for Crohn’s Disease (SES-CD) included in the dataset were sorted. The SPSS software (v26.0; IBM Corp., Armonk, NY) was used to analyze the age and SES-CD data as continuous variables to obtain the mean and standard deviation. The “rstatix” (v0.7.0), “ggplot2,” and “ggpubr” data packages in the R software were used to analyze sex, age, lesion range, smoking status, SES-CD, and clinical characteristics between subgroups using one-way analysis of variance to identify the basic features of each subgroup.

### 2.4. Screening differentially expressed genes (DEGs) in the subgroups

Gene expression levels were compared between the subgroups and between each subgroup and the control group. The “limma” package in the R software was used to screen for DEGs. These were identified as those with a |log fold change| >.2 and an adjusted *P* value < .05; the value was adjusted using the Benjamin–Hochberg method. Through comparison, we identified genes that were significantly upregulated in only one subgroup and labeled these as the specific genes of that subgroup.

### 2.5. Gene set enrichment analysis (GSEA) of subgroups

The Perl software (v5.32.1; https://www.perl.org/) was used to convert the datasets into gene lists and gene set files for each subgroup. These files were transferred to the GSEA software (v4.1.0; https://www.gsea-msigdb.org/gsea/msigdb/), and the maximum running value was set to 5000 to run large amounts of data.

### 2.6. Weighted gene co-expression network analysis (WGCNA)

The “WGCNA” (v1.71) package in the R software was used to analyze the genes in the dataset.^[15]^ In this study, the WGCNA data package was used to cluster genes with highly similar expression patterns and to match the clustering results with the corresponding clinical features. Samples were clustered to exclude outliers. The pickSoftThreshold function was then used to calculate the soft threshold, allowing a scale-free network to be constructed. The correlation values between DEGs and the correlation matrix were calculated using the power function method. Using the optimal soft threshold, an adjacency matrix was generated for the DEGs and was then transformed into a topological overlap matrix. Using the dynamic tree method and different matrix degrees, different gene tree diagrams were generated from the topological overlap matrix to obtain gene modules.^[15]^ Finally, a regression analysis of clinical characteristics and gene expression was performed using *P* values, and the relationship between the Pearson correlation analysis module was used to analyze the clinical characteristics.

### 2.7. Functional enrichment analysis of genes

The “clusterProfiler” (v.4.6.2), “org.Hs.e.g.db” (v.3.16.0), and “enrichplot” (v.1.18.3) packages in the R software were used for the Gene Ontology (GO) and Kyoto Encyclopedia of Genes and Genomes (KEGG) analyses of CD-related DEGs.^[16]^ The pathway with the most significant difference in each gene module was selected, and the relationship between the pathway and subgroup was analyzed using Pearson correlation.

### 2.8. Statistical analysis

SPSS was used to obtain the mean ± standard deviation of age and SES-CD, and the chi-square test was used to analyze differences in the smoking status, sex, and pathological range. The R software (v4.2.2) uses the x86_64-pc-linux-gnu (64 bit) platform. Results from two-tailed tests with *P* < .05 were considered statistically significant. Pearson correlation coefficients were used to analyze the correlation between gene modules and subgroups.

## 3. Results

### 3.1. Data processing and grouping

**Figure 1.**
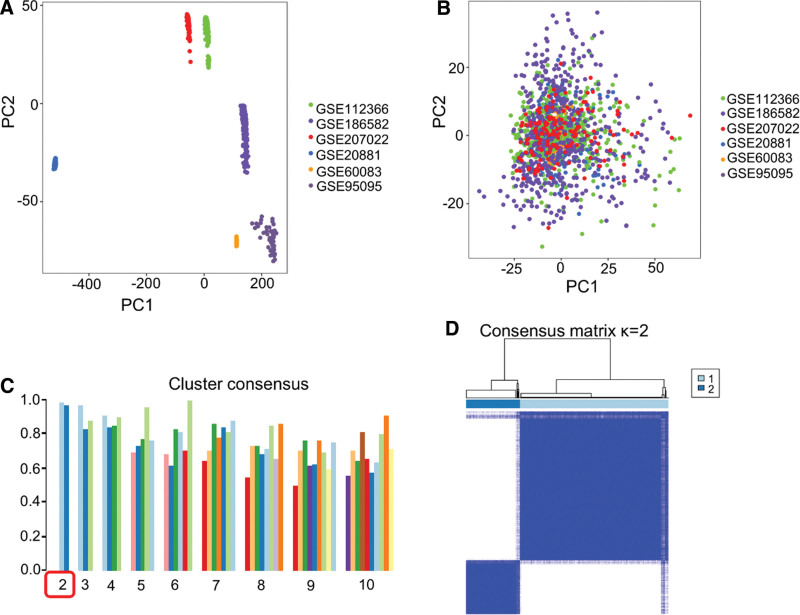
Processing and grouping of transcriptome data. (A) Individual datasets before batch-effect elimination. (B) Overall dataset after batch-effect elimination. (C) Consistency evaluation of 9 categories using unsupervised learning. (D) Cluster correlation heat map after division into 2 subgroups.

To eliminate the batch effect between datasets, the GSE112366, GSE186582, GSE207022, GSE20881, GSE60083, and GSE95095 datasets were processed to obtain a normalized overall dataset, such that the data could be visualized using principal component clustering. The results showed an absence of correlation between the datasets before preprocessing (Fig. 1A). After processing, the samples were uniformly concentrated, and the obtained dataset was used for all subsequent analyses (Fig. 1B).

A total of 1156 CD and 201 control group samples were included in this study. Using unsupervised learning, 9 subgroups were obtained that were evaluated for consistency. After evaluation, all samples were divided into 2 subgroups, each with a consistency score close to 1.0, higher than that of other subgroups, indicating high gene aggregation and expression similarity within each group (Fig. 1C). In addition, the clustering heat map results for the subgroups showed that the density within each group of the 2 subgroups was high and that there was no correlation between the groups (Fig. 1D).

### 3.2. Clinical characteristics of the subgroups

**Figure 2.**
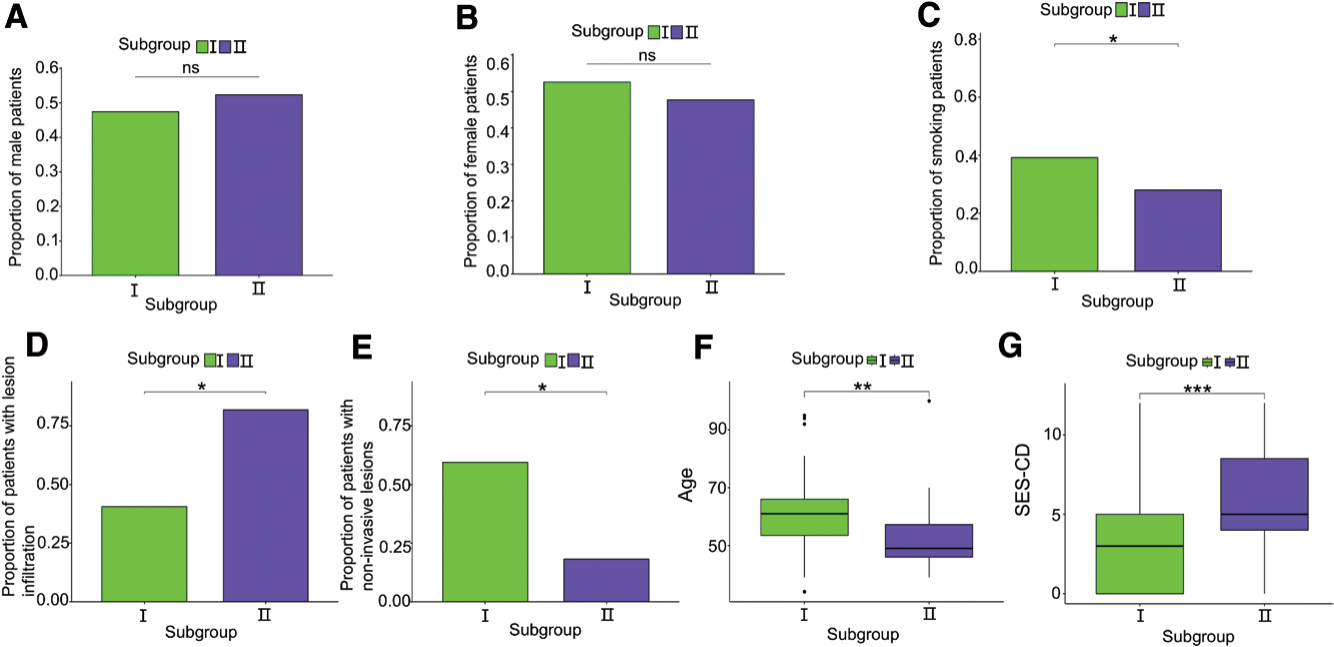
Clinical characteristics of subgroups. (A) Proportion of male patients in each subgroup. (B) Proportion of female patients in each subgroup. (C) Proportion of smokers in each subgroup. (D) Proportion of patients with lesion infiltration in each subgroup. (E) Proportion of patients with noninvasive lesions in each subgroup. (F) Patient age for each subgroup. (G) SES-CD in each subgroup. *P < .05, **P < .01, ***P < .001. ns = no significant difference, SES-CD = Simple Endoscopic Score for Crohn’s Disease.

To understand the clinical characteristics of each subgroup, we sorted the patients by sex, age, smoking status, whether the lesion was infiltrated, and SES-CD included in the dataset. There was no significant difference in the number of male and female patients between the 2 subgroups (Figs. 2A and B). In subgroup I, the number of smokers was considerably higher than that in subgroup II (Fig. 2C). There were significantly more patients with infiltrating lesions (Fig. 2D), and hence, fewer patients without infiltrating lesions (*P < *.05; Fig. 2E) in subgroup II than in subgroup I. Analysis using the SPSS software showed that the mean ± standard deviation of patients’ age was 60.39 ± 12.492 and 53.64 ± 14.198 years, while that of SES-CD was 2.73 ± 3.154 and 5.83 ± 3.442, for subgroups I and II, respectively. Patients in subgroup I were significantly older than those in subgroup II (*P* *<* .01; Fig. 2F), and the SES-CD was also significantly different between the 2 subgroups (*P* *<* .001; Fig. 2G).

### 3.3. Subgroup-specific genes

**Figure 3.**
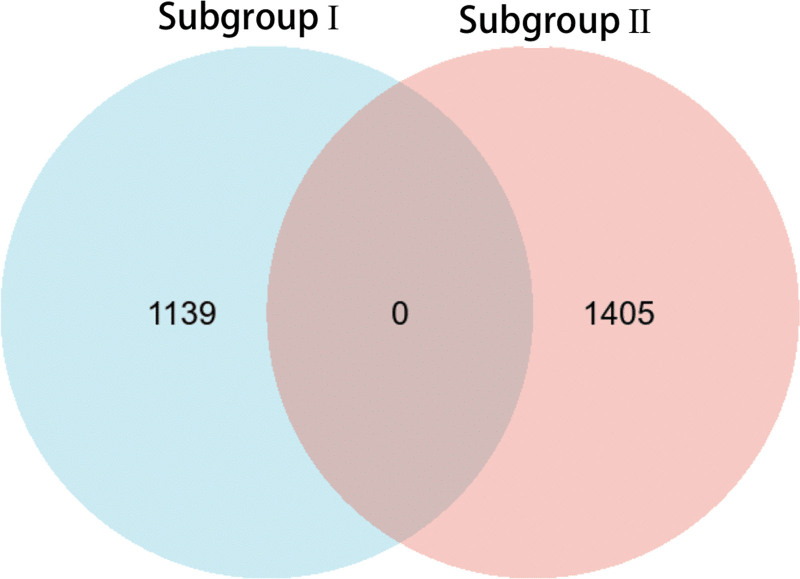
Subgroup-specific gene intersection results. There are no common genes in the intersection.

We analyzed the molecular characteristics of the subgroups and screened for specific genes. Upon comparing gene expression patterns between the subgroups and between each subgroup and the control group, we identified 1139 and 1456 genes specific to subgroups I and II, respectively. Table 1 lists the top 10 genes in each subgroup. To determine whether there were genes common to each subgroup, nonidentical genes were identified by crossing the gene groups (Fig. 3).

**Table 1:** The top 10 specifically upregulated differentially expressed genes in each subgroup.

Subgroup I	Subgroup II
*CDKN2B*	*CD44*
*SLC22A5*	*WARS*
*PHYH*	*PIM2*
*PXMP2*	*FGR*
*MAOA*	*CTSK*
*ABCD3*	*PTAFR*
*VIPR1*	*IL10RA*
*ACOX1*	*GBP5*
*SCIN*	*SELL*
*SMPDL3A*	*RAB31*

### 3.4. GSEA

**Figure 4.**
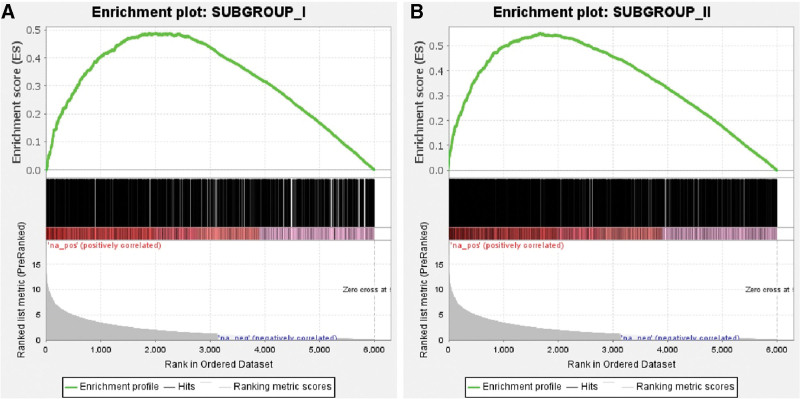
GSEA of subgroups I (A) and II (B). The green line indicates the enrichment score, each black line represents a gene, and the gray area reflects the signal-to-noise ratio between the subgroup and control group. GSEA = gene set enrichment analysis.

GSEA was used to determine the overall differences in DEGs that are specific to each subgroup and between subgroups and the control group. As observed in Fig. 4A and B, most DEGs in the 2 subgroups are concentrated on the left-hand side of the image, with both the *P* value and error detection rate being <.01. These results indicate that the uniqueness of the DEGs between subgroups and between subgroups and the control group was the same.

### 3.5. WGCNA

**Figure 5.**
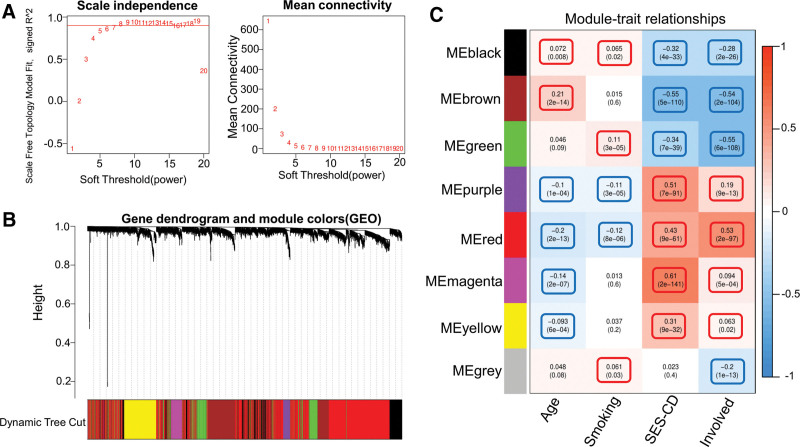
WGCNA results. (A) The left-hand side graph shows the analysis of the scale-free fitting index (*y*-axis) and power value (*x*-axis). The right-hand side graph shows the analysis of the connectivity (*y*-axis) and power value (*x*-axis). (B) Different gene modules obtained by dynamic tree analysis. (C) Heat map of the relationship between different gene modules and clinical characteristics. The red and blue colors represent a significant upregulation and downregulation of gene expression, respectively. GEO = Gene Expression Omnibus, SES-CD = Simple Endoscopic Score for Crohn’s Disease, WGCNA = weighted gene co-expression network analysis.

**Figure 6.**
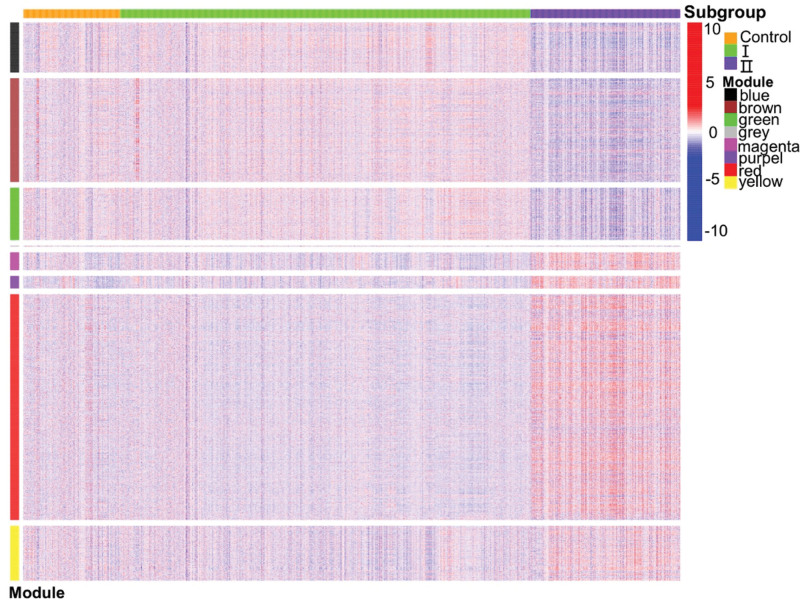
Heat map of the relationship between subpopulations and color modules. Red and blue represent upregulation and downregulation of gene expression, respectively.

In total, 5996 genes were included in the WGCNA. Gene similarity was consistent with a scale-free network, and the optimal output power value was 7 (Fig. 5A). The dynamic tree analysis revealed 8 differently colored gene modules (Fig. 5B): black, brown, green, purple, red, magenta, yellow, and gray. The Pearson correlation between the color modules and clinical characteristics showed that age was significantly and positively correlated with the black (*P* = .008) and brown (*P* = 2e−18) modules and significantly and negatively correlated with the purple (*P* = 1e−04), red (*P* = 2e−13), magenta (*P* = 2e−07), and yellow (*P* = 6e−04) modules. Smoking was positively correlated with the black (*P = *.02), green (*P* = 3e−05), and gray (*P* = .03) modules and negatively correlated with the purple (*P* = 3e−05) and red (*P* = 8e−06) modules. SES-CD was negatively correlated with the black (*P* = 4e−33), brown (*P* = 5e−110), and green (*P* = 7e−39) modules and positively correlated with the purple (*P* = 7e−91), red (*P* = 9e−61), magenta (*P* = 2e−141), and yellow (*P* = 9e−32) modules. Lesion infiltration was negatively correlated with the black (*P* = 2e−26), brown (*P* = 2e−104), green (*P *= 6e−108), and gray (*P* = 1e−13) modules and positively correlated with the purple (*P* = 9e−13), red (*P* = 2e−97), magenta (*P* = 5e−04), and yellow (*P* = .02) modules (Fig. 5C). Correlation analysis of subgroups and color modules revealed that the black, brown, and green modules were more enriched in subgroup I than in subgroup II, and the other modules were more enriched in subgroup II than in subgroup I (Fig. 6).

### 3.6. GO and KEGG pathway enrichment analyses

**Figure 7.**
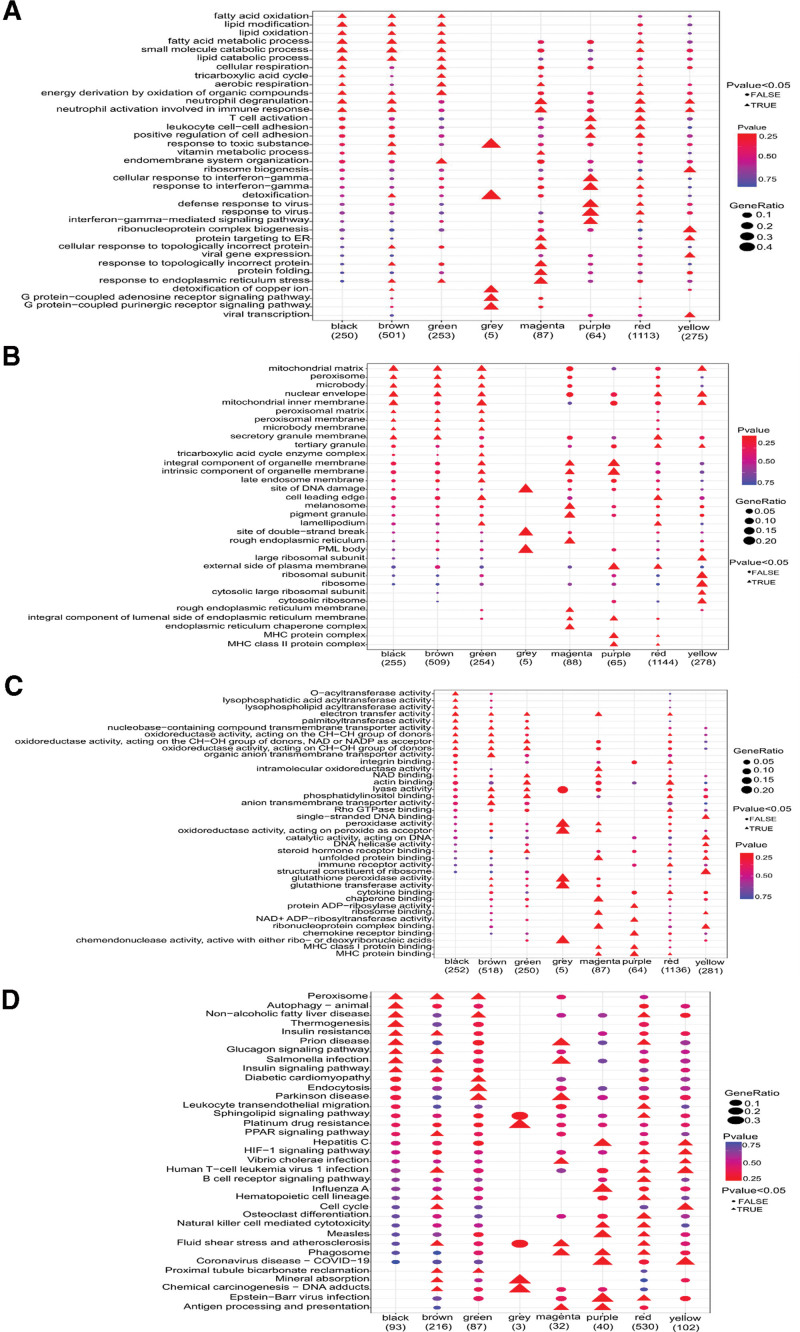
GO and KEGG pathway enrichment analyses. (A) BP enrichment results. (B) CC enrichment results. (C) MF enrichment results. (D) KEGG pathway enrichment results. The triangles and circles indicate significant and nonsignificant enrichment, respectively. The larger the shape, the higher the level of gene enrichment. The red and blue colors indicate low and high *P* values, respectively. BP = biological process, CC = cellular component, EB = Epstein–Barr, GO = Gene Ontology, HIF-1 = hypoxia-inducible factor 1, KEGG = Kyoto Encyclopedia of Genes and Genomes, MF = molecular function, MHC = major histocompatibility complex, PML = promyelocytic leukemia.

To understand the biological characteristics of the subpopulations, GO analysis was performed. In the biological process category, fatty acid oxidation and lipid catalytic processes were mainly concentrated in the black, brown, and green modules; by contrast, T cell activation, leukocyte cell–cell adhesion, positive regulation of cell adhesion, cell response to interferon-gamma, defense response to virus, protein targeting to the endoplasmic reticulum, and virtual gene expression were mainly enriched in the magenta, purple, red, and yellow modules (Fig. 7A). In the cellular component category, peroxisomes, and microbodies were mainly concentrated in the black, brown, and green modules, whereas the external side of the plasma membrane, ribosomes, endoplasmic reticulum, and major histocompatibility complex protein complexes were mainly concentrated in the purple and red modules (Fig. 7B). In the molecular function category, nuclease-containing compound transporter activity, oxidoreductase activity, lyase activity, and peroxidase activity were mainly concentrated in the black, brown, and green modules; however, ribosome binding and major histocompatibility complex protein binding were mainly concentrated in the magenta, purple, red, and yellow modules (Fig. 7C).

**Figure 8.**
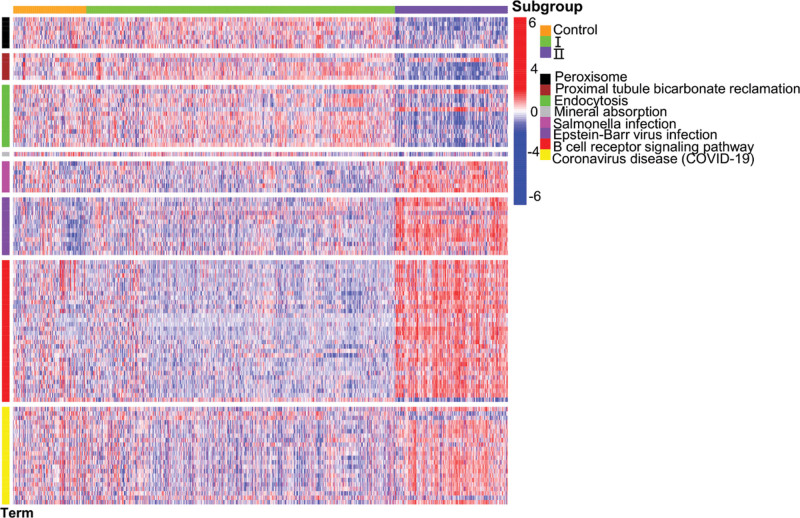
Heat map of the relationship between KEGG pathways and subgroups. KEGG = Kyoto Encyclopedia of Genes and Genomes.

The KEGG pathway analysis results showed that the peroxisome, insulin signaling pathway, peroxisome proliferator-activated receptor signaling pathway, proximal tube bicarbonate regeneration, and chemical carcinogenesis were mainly concentrated in the black, brown, and green modules; by contrast, hepatitis C, hypoxia-inducible factor 1 signaling pathway, *Vibrio cholerae* infection, influenza A, natural killer cell-mediated cytotoxicity, measles, phagosome, coronavirus disease 2019 (COVID-19), Epstein–Barr virus (EBV) infection, and antigen processing and presentation were mainly concentrated in the magenta, purple, red, and yellow modules (Fig. 7D). The analysis of the relationships between the most considerably enriched pathways and subgroups in each color module showed that peroxisomes, proximal tube bicarbonate regeneration, endocytosis, and mineral absorption were positively correlated with subgroup I. *Salmonella* infection, EBV infection, B cell receiver signaling pathway, and COVID-19 were negatively correlated with subgroup I, whereas subgroup II showed the opposite result (Fig. 8).

### 3.7. Summary of subgroup characteristics

A summary of the clinical, molecular, biological, and therapeutic characteristics of each subgroup is presented in Table 2. The results demonstrate that the subgroups can be used to diagnose and treat patients with CD.

**Table 2:** Summary of the characteristics of the subjects from each subgroup.

Item	Characteristics
Subgroup I	
Clinical features	Smoking status (yes), age (older), SES-CD (low), disease extent (no lesion infiltration)
Specific genes	*CDKN2B, SLC22A5, PHYH, PXMP2, MAOA, ABCD3, VIPR1, ACOX1, SCIN, SMPDL3A*
BP	Fatty acid oxidation, lipid catabolic process, response to toxic substance, detoxification of copper ion, G proteincoupled adenosine receptor signaling pathway
CC	Mitochondrial matrix, peroxisome, microbody
MF	Nucleobase-containing compound transmembrane transporter activity, oxidoreductase activity, acting on the CH−CH group of donors, lyase activity, glutathione peroxidase activity
KEGG	Peroxisome, insulin signaling pathway, glucagon signaling pathway, platinum drug resistance, proximal tubule bicarbonate reclamation, mineral absorption, chemical carcinogenesis-DNA adducts
Effective drugs	Treatment of dysbacteriosis (rifampin, rifaximin, or GIT2-targeted drugs)
Subgroup II	
Clinical features	Smoking status (no), age (younger), SES-CD (high), disease extent (lesion infiltration)
Specific genes	*CD44, WARS, PIM2, FGR, CTSK, PTAFR, IL10RA, GBP5, SELL, RAB31*
BP	T cell activation, leukocyte cell–cell adhesion, positive regulation of cell adhesion, response to interferon-gamma, response to virus, interferon-gamma-mediated signaling pathway, protein targeting to endoplasmic reticulum
CC	Tertiary granule, external side of plasma membrane, integral component of lumenal side of endoplasmic reticulum membrane, MHC protein complex
MF	Single-stranded DNA binding, structural constituent of ribosome
KEGG	Hepatitis C, hypoxia-inducible factor 1 signaling pathway, *Vibrio cholerae* infection, Influenza A, natural killer cellmediated cytotoxicity, measles, phagosome, coronavirus disease 2019, Epstein–Barr virus infection, antigen processing and presentation
Effective drugs	Antiviral therapy

BP = biological process, CC = cellular component, KEGG = Kyoto Encyclopedia of Genes and Genomes, MF = molecular function, MHC = major histocompatibility complex, SES-CD = Simple Endoscopic Score for Crohn’s Disease.

## 4. Discussion

Currently, CD cannot be eradicated, and specific drugs or treatment programs can only alleviate the disease. Based on the transcriptome data from patients with CD, we used an unsupervised clustering method to divide the patients into 2 subgroups. The clinical characteristics of and molecules specific to each subgroup were identified using the R software, SPSS software, and other statistical tools. The overall characteristics of the subgroups were described in combination with GO and KEGG pathways. This study provides a theoretical basis for exploring pathogenesis-related genes and therapeutic targets in CD.

In recent years, with the development of molecular medicine, the application of molecular science in the context of CD has progressed.^[17,18]^ Bioinformatics analysis is a useful tool for exploring the pathogenesis of diseases and screening for new therapeutic targets. A previous study identified no sex-specific differences in adult patients with CD,^[2]^ a finding also seen in the present study, with no significant difference observed in the sex of patients between the subgroups. Therefore, sex cannot be used as a basis for identifying different molecular subgroups.

Patients with a history of smoking were present primarily in subgroup I, and based on the results of disease infiltration, the condition of subgroup II patients was more serious than that of subgroup I patients. Endoscopy is an important tool for disease or treatment effect evaluation, and many ongoing biological agent trials require endoscopic evaluation.^[19]^ SES-CD was developed as a simple and rapid CD endoscopic scoring system and was verified in the present study^[20]^; the higher the SES-CD, the more severe the condition. In this study, the SES-CD in subgroup II was significantly higher than that in subgroup I, which coincided with the results of disease infiltration, showing that the condition of patients in subgroup II was more serious.

Cyclin-dependent kinase inhibitor 2B (*CDKN2B*) is associated with various types of cancer, heart disease, and inflammatory diseases.^[21]^ Three tumor suppressor genes, *p14^ARF^, p16^INK4A^*, and *p15*^*INK4B*^, are encoded within the *CDKN2B* locus and participate in the retinoblastoma and p53 pathways.^[22]^ CDKN2B regulates the cell cycle in tumors and has a destructive effect on cancer.^[23]^ The abnormal expression of the long-chain noncoding RNA cyclin-dependent kinase inhibitor 2B antisense RNA 1 has been detected in human colorectal cancer (CRC), which can inhibit the proliferation and migration of CRC cells and promote cell apoptosis by activating the MEK/ERK/p38 signaling pathway.^[24]^ Among patients with CD, the incidence rate of CRC in 30 years is 8.3%.^[25]^ We found *CDKN2B* to be the most subgroup I-specific gene; therefore, it is inferred that the risk of CRC in subgroup I patients will be lower than that in subgroup II patients.

The specific gene, interleukin 10 receptor, alpha subunit, in subgroup II codes for a cytokine that inhibits intestinal inflammation. The IL-10/signal transducer and activator of transcription 3 (STAT3) signaling pathway plays an important role in controlling inflammation and protecting the intestinal tissue from inflammatory invasion.^[26]^ During inflammation, IL-10 binds to receptor A/B (IL-10RA/B), activates Jak1 and Tyk2, and induces STAT3 phosphorylation. STAT3 translocates into the nucleus to regulate the transcription of related genes and promotes an anti-inflammatory response.^[27]^ CD occurs in mice with *Il10* knockout.^[28]^ Young patients with an IL-10 or IL-10R deficiency may develop severe infantile colitis, similar to CD.^[29]^ Therefore, *IL10* and *STAT3* have been characterized as CD-related genes.^[30]^

Intestinal microbiota disorders play a key role in the occurrence and development of inflammatory bowel disease, and genetic defects in innate immunity are predisposing factors for microbiota disorders.^[31]^ The imbalance of intestinal flora, especially an increase in the abundance of members of the family *Enterobacteriaceae*, triggers an adherent and invasive *Escherichia coli* (AIEC) pathotype.^[32]^ AIEC can use inflammation-related substances to grow, invade, or penetrate the intestinal mucosa through flagella and hairy adhesins (such as FimH and LpFA) and enter macrophages. AIEC can continue to exist, proliferate in macrophages, and intensify inflammation.^[33]^ There is a close association between dysfunctional enterobacteria and celiac disease. Therefore, the use of antibiotics targeting abnormal microbiota can effectively treat celiac disease. Rifampicin has good antibacterial activity against gram-positive, gram-negative, aerobic, and anaerobic bacteria, and can ameliorate the symptoms of patients with mild to moderate celiac disease.^[31,34]^ G protein-coupled receptor kinase-interactor 2 (GIT2) belongs to the adenosine diphosphate ribosylation factor-directed GTPase-activated protein family.^[35]^ GIT2 is highly expressed in monocytes and macrophages, particularly after antigen stimulation.^[36]^ In mouse models of severe acute and chronic CD, *Git2* knockout was found to produce mice that were more susceptible to *E coli* infection and endotoxin shock.^[37]^ GIT2 can inhibit the inflammatory response by regulating the role of the toll-like receptor signaling pathway in the intestinal immune response and is considered the key terminator of the toll-like receptor-induced inflammatory response. Therefore, GIT2 is a potential target for the treatment of CD.

GO and KEGG pathway analyses showed that various virus-related biological characteristics or pathways, including those relating to hepatitis C, measles, COVID-19, and EBV infection, were significantly enriched in subgroup II. Studies have confirmed that in patients with CD, viral infections can cause inflammatory reactions according to the genetic background, resulting in abnormal intestinal microflora and disruption of the normal microflora environment.^[38]^ Harley et al found that EBV gene products (EBNA2) are related to gene–environment interactions and inferred that EBV plays a role in the pathogenesis of CD.^[39]^ In addition, cytomegalovirus and herpesvirus immunoglobulin G were detected in a 23-year-old patient.^[40]^ To summarize, an increasing number of studies have shown that viruses play a role in CD pathogenesis, and, thus, it may be possible to target the virus to treat CD.

Overall, we used a gene expression clustering method to classify patients with CD and analyzed subgroups based on their clinical and molecular characteristics. This study has several limitations in terms of disease classification. In particular, the molecular subtypes established in this study must be verified using proteomics, metabonomics, and genomics methodologies. In addition, future studies should include a larger population and analyze more detailed clinical information. Overcoming these challenges would further aid in the development of precision therapy for CD.

## 5. Conclusions

In summary, patients with CD were divided into 2 subgroups using a bioinformatics approach. Compared to subgroup II, subgroup I predominantly included patients who smoked, who were older, and who showed a limited lesion range; the genes specific to this subgroup were *CDKN2B*, solute carrier family 22 member 5, and phytanoyl-CoA 2-hydroxylase, among others, which may be effective in treating dysbacteriosis. Compared to those in subgroup I, the patients in subgroup II were younger and had more serious disease manifestations; the specific genes identified were cluster of differentiation 44, tryptophanyl-tRNA synthetase, and interleukin 10 receptor, alpha subunit, among others, which may be effective in antiviral treatment. The establishment of molecular subgroups provides a basis for the development of new diagnostic methods and individualized therapeutic strategies for patients with CD.

## Author contributions

**Formal analysis:** Jianglei Ma.

**Methodology:** Jianglei Ma, Huijie Zhang.

**Software:** Jianglei Ma, Huijie Zhang.

**Writing – original draft:** Jianglei Ma, Huijie Zhang.

**Data curation:** Huijie Zhang.

**Conceptualization:** Yuanyuan Zhang, Guangming Wang.

**Writing – review & editing:** Yuanyuan Zhang, Guangming Wang.

**Funding acquisition:** Guangming Wang.
